# Association of chronic obstructive pulmonary disease with risk of lung cancer in individuals aged 40 years and older: A cross-sectional study based on NHANES 2013–2018

**DOI:** 10.1371/journal.pone.0311537

**Published:** 2024-10-23

**Authors:** Hong Chen, Xiao-Bo Hu, Jin Zhou, Chen-Yun He, Ke Wang, Qun Yi

**Affiliations:** 1 Department of Respiratory and Critical Care Medicine, Chengdu Second People’s Hospital, Chengdu, China; 2 Department of Medical Oncology, Sichuan Clinical Research Center for Cancer, Sichuan Cancer Hospital & Institute, Sichuan Cancer Center, Affiliated Cancer Hospital of University of Electronic Science and Technology of China, Chengdu, China; 3 Department of Respiratory and Critical Care Medicine, West China Hospital, Sichuan University, Chengdu, China; Kyung Hee University School of Medicine, REPUBLIC OF KOREA

## Abstract

**Background:**

It remains unclear whether chronic obstructive pulmonary disease (COPD) is an independent risk factor for lung cancer after excluding confounding factors such as smoking, age, sex, body mass index (BMI), comorbidities, etc.

**Methods:**

Data from 11,440 participants (≥ 40 years old) in the National Health and Nutrition Examination Survey (NHANES) 2013–2018 were analyzed. Weighted multivariable logistic regression models were used to assess the association between COPD and lung cancer risk. Subgroup analyses were based on age, sex, BMI, and smoking.

**Results:**

This study included 660 patients with COPD and 10,780 participants without COPD. The prevalence of lung cancer was significantly higher in patients with COPD compared to participants without COPD (3.39% vs 0.14%). After adjusting for confounding factors, COPD was associated with a significantly increased risk of lung cancer (OR, 12.24, 95% CI, 4.99–30.06, p < 0.001). This association remained significant in all subgroups, particularly in individuals aged > 65 years (OR, 20.05, 95% CI, 6.85–58.72, p < 0.001), smokers (OR, 19.38, 95% CI, 2.02–185.66, p = 0.010), males (OR, 17.39, 95% CI, 5.28–57.31, p < 0.001), individuals who quit smoking within 10 years (OR, 12.86, 95% CI, 2.59, 63.99, p = 0.002), and individuals with a BMI > 25 kg/m^2^ (OR, 14.56, 95% CI, 3.88–54.69, p < 0.001).

**Conclusions:**

COPD is an independent risk factor for lung cancer, especially in certain subgroups. The combination of COPD and smoking greatly amplifies the lung cancer risk. These findings highlight the importance of early lung cancer screening in patients with COPD.

## Introduction

Globally, chronic obstructive pulmonary disease (COPD) and lung cancer are both leading causes of death, particularly in individuals over 40. Lung cancer is one of the most common and deadliest malignancies worldwide, while COPD ranks third as a cause of mortality [1–3]. The two diseases exhibit high morbidity, especially among smokers, with even higher incidence rates [4, 5]. This may be attributed to common risk factors such as smoking, air pollution, occupational exposure, and advanced age. Moreover, they share common mechanisms, including chronic inflammation of the airways, genetic susceptibility, abnormal immune responses, oxidative stress, etc [6, 7]. Clinically, both diseases share similar symptoms such as chronic cough, sputum production, and dyspnea. However, the presence of COPD can adversely affect anti-tumor therapy and reduce the survival benefits of patients, including limiting surgical opportunities, increasing postoperative complications, prolonged oxygen therapy and hospital stay, reducing benefits from chemotherapy and radiotherapy, lower epidermal growth factor receptor (EGFR) mutation rates, and worse efficacy after targeted therapy with EGFR-tyrosine kinase inhibitors (EGFR-TKI) [8–11]. In advanced lung cancer patients with severe COPD, even distinguishing the cause of death as either lung cancer or COPD is challenging. Therefore, early screening for lung cancer in patients with COPD is of significant importance.

Studies have reported a significant increase in lung cancer risk among patients with COPD [12–14]. However, limited studies exist concerning whether COPD is an independent risk factor for lung cancer, especially after excluding confounding factors such as smoking, age, sex, body mass index (BMI), comorbidities, etc. Especially, extensive evidence indicates that smoking is the primary risk factor for both lung cancer and COPD. On the one hand, smoking is an overwhelming strong causative factor of lung cancer, long-term smokers have 10 to 30 times the risk of lung cancer compared to non-smokers [15–17]. On the other hand, epidemiological studies suggest that smoking is also the most crucial risk factor for COPD. A retrospective cohort study (n = 8,045) found that individuals who smoked throughout the 25-year observation period are more likely to develop COPD (36% vs. 8%) than nonsmokers [18]. However, apart from smoking, other contributing factors are implicated in the development of these two diseases, with non-smokers accounting for approximately 25% of lung cancer and nearly 30% of COPD [19]. In addition, recent studies suggest that COPD has different phenotypes, requiring further studies to identify subgroups of patients with COPD with significantly increased lung cancer risk to allow early and frequent screening and monitoring of lung cancer risks so that these patients can receive timely and effective treatment.

Therefore, this study aimed to assess the association between COPD and lung cancer prevalence among individuals aged 40 and older, based on data collected from the National Health and Nutrition Examination Survey (NHANES), with an additional focus on the differential risk of lung cancer among subgroups of patients with COPD.

## Materials and methods

### Study population and data source

**Fig 1:**
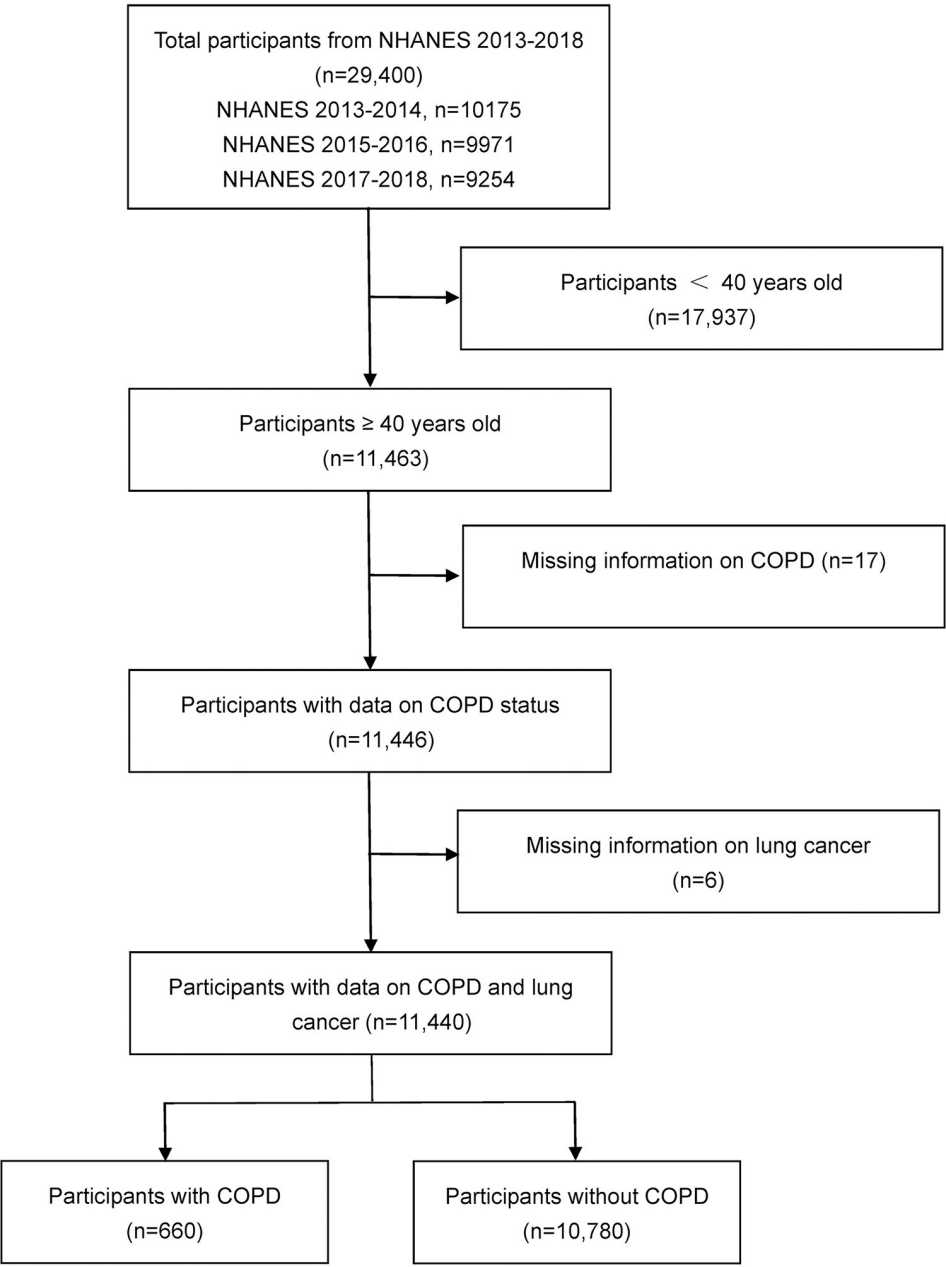
Flow chart of the study population. NHANES, National Health and Nutrition Examination Survey; COPD, chronic obstructive pulmonary disease.

Data (publicly accessible globally) analyzed in our study were obtained from the NHANES. The National Center for Health Statistics (NCHS) Ethics Review Board granted approval for the conduct of NHANES and written informed consents were obtained from all participants. As no ethical approval is required to access and analyze the publicly available, de-identified NHANES database, exemption was confirmed by the Chengdu Second People’s Hospital Institutional Review Board. NHANES aims to assess the health and nutrition status of adults and children in the United States. It employs a stratified multi-stage probability cluster design and conducts cross-sectional surveys every two years (an NHANES cycle) to provide a representative sample of health information for the US non-institutionalized civilian population, forming a large health database. The database includes demographic data, dietary data, laboratory data, examination data, questionnaire data, and limited access data. All data collected by NHANES are used to determine the prevalence of major diseases and their corresponding risk factors and serve as the basis for national standards on height, weight, and blood pressure. Notably, the questionnaires employed in NHANES undergo rigorous design and standardization processes, ensuring the use of consistent survey instruments across each cycle. The health status of participants, such as whether they have lung cancer, COPD, etc., is obtained through these questionnaires. The conditions are assessed simultaneously, as the survey captures the participants’ health status at the same point in time. Consequently, association analyses based on NHANES data mitigate the potential for overestimating the association between lung cancer and related risk factors, as they avoid the bias introduced by more extensive investigations among lung cancer patients. More details about the NHANES study, sampling methods, and design have previously been published. This study included three NHANES cycles (2013–2014, 2015–2016, and 2017–2018) as only these three cycles simultaneously provided information on whether participants have COPD and lung cancer. In the original data, there were a total of 29,400 participants. Since the prevalence of COPD is extremely low in individuals under 40 years old, and the diagnosis of COPD may be unreliable in this age group for asthma cannot be excluded, our study only included data of individuals aged 40 years and older. In the final study population, we evaluated data from 11,440 adults aged ≥ 40 years old, including 660 patients with COPD and 10,780 participants without COPD (**Fig 1**). The exposure variable is whether a participant had COPD, determined by their response to whether a doctor had informed them that they had COPD. Similarly, the outcome variable is based on whether a doctor had informed them that they had lung cancer. Participants who answered ’yes’ were included in the respective disease group, while those who answered ’no’ were included in the control group. Participants with missing data were excluded. These criteria and data sources are detailed in **S1 Table in S1 File**, which also includes the relevant questionnaire items in the NHANES questionnaire data (Medical Conditions section) (e.g., MCQ160o - ’Ever told you had COPD?’).

### Covariate evaluation

Studies have been conducted in recent decades to identify the risk factors associated with lung cancer. Personal and environmental variables, such as advanced age, smoking, and comorbidities, have been found to be linked to an increased risk of lung cancer. To minimize potential bias on study results due to these confounding factors, adjustments were made for confounding factors (covariates) in the statistical models. This study examined three categories of covariates, including baseline demographic data of participants (age, sex, race, BMI, and smoking status including smoking history and cessation duration), socioeconomic status (annual family income and education level), and comorbidities (asthma, chronic bronchitis, emphysema, diabetes, chronic heart failure, coronary heart disease, and stroke). These variables were extracted from the demographics data, laboratory data, examination data, and questionnaire data of the NHANES database, and their respective measurement methods can be accessed on the NHANES website. The data source for these covariates are shown in **S1 Table in S1 File**.

### Subgroup analyses

In order to further explore the lung cancer risk of different subgroups of patients with COPD and their relative risk compared to participants without COPD, we conducted subgroup analyses. These subgroup analyses were based on age (**≤** 65 years old or > 65 years old), sex (male or female), BMI (**≤** 25 kg/m^2^ or > 25 kg/m^2^), and smoking status, including smoking history (smokers or non-smokers) and smoking cessation duration (**≤** 10 years or>10 years).

### Statistical analysis

We conducted statistical analyses based on the analysis guidelines of the NHANES database. Statistical analysis was performed using Stata software (version 17). All data analysis in this study was conducted using NHANES sample weights, as NHANES aims to generate representative data of the civilian noninstitutionalized American population. As three cycles of NHANES data were included, the sample weight (WTMEC6YR) was calculated as WTMEC6YR = WTMEC2YR × 1/3 following NHANES recommendations. Descriptive data were presented as means ± standard deviations (SD) for continuous variables or as proportions for categorical variables. Weighted multivariable logistic regression models were employed to investigate the association between COPD and the risk of lung cancer. Odds ratios (ORs) with corresponding 95% confidence intervals (CIs) were presented. In accordance with the STrengthening the Reporting of OBservational studies in Epidemiology (STROBE) statement [20], three models were conducted to account for covariates and determine whether COPD is an independent risk factor for lung cancer: an unadjusted model with no covariate adjustment (unadjusted OR), a minimally adjusted model with adjustment for age, sex, and race (minimally adjusted OR), and a fully adjusted model with adjustment for all analyzed covariates (fully adjusted OR), including baseline demographic characteristics, lifestyle factors, and comorbidities. All p-values were two-tailed, and a statistical significance level of 0.05 was adopted.

## Results

### Characteristics of participants

This study analyzed data from 11,440 participants aged 40 years and older in NHANES 2013–2018, including 660 patients with COPD and 10,780 participants without COPD. Patients with COPD and participants without COPD showed significant differences in baseline demographic characteristics, socioeconomic status, and comorbidities based on the weighted survey data (**Table 1**). Regarding baseline demographic characteristics, compared with participants without COPD, patients with COPD were older (64.46 years old vs 58.17 years old), had a higher proportion of individuals aged ≥ 65 years old (43.99% vs 28.16%), non-Hispanic Whites (79.52% vs 67.71%), smokers (46.22% vs 14.83%), and smoking cessation duration ≤ 10 years (16.44% vs 8.03%). In terms of socioeconomic status, patients with COPD had lower overall education levels (< high school: 21.47% vs 13.99%) and annual family income (< 20000 USD: 34.26% vs 13.16%). Moreover, comorbidities were more common in patients with COPD: chronic bronchitis, 41.93% vs 5.41%; emphysema, 35.31% vs 0.62%; asthma, 40.68% vs 12.45%; diabetes, 29.54% vs 15.02%; chronic heart failure, 18.76% vs 2.83%; coronary heart disease, 23.49% vs 4.70%; stroke, 14.21% vs 3.91%.

**Table 1:** Characteristics of the study population from NHANES 2013–2018 (N = 11,440).

Characteristic	Participants with COPD	Participants without COPD	*P* value
**Number of participants**	660	10780	
**Age (years)**	64.46 ± 10.26	58.17 ± 11.71	<0.001
**Age (%)**			<0.001
** ≤ 65 years old**	56.01	71.84	
** > 65 years old**	43.99	28.16	
**Sex (%)**			0.842
** Male**	47.67	47.26	
** Female**	52.33	52.74	
**Race (%)**			<0.001
** Mexican American**	2.03	7.41	
** Other Hispanic**	2.13	5.41	
** Non-Hispanic white**	79.52	67.71	
** Non-Hispanic black**	7.77	10.83	
** Other Race**	8.55	8.64	
**Body mass index (kg/m** ^**2**^ **)**	30.05 ± 7.86	29.76 ± 6.69	0.304
**Body mass index (%)**			<0.0001
** ≤ 25 kg/m** ^**2**^	25.81	24.26	
** > 25 kg/m** ^**2**^	70.67	74.50	
** Unclassified**	3.52	1.25	
**Smoking (%)**			
**Smoking history**			<0.001
** Smokers**	46.22	14.83	
** Non-smokers**	41.38	28.91	
** Unclassified**	12.40	56.26	
**Smoking cessation duration**			<0.001
** ≤ 10 years**	16.44	8.03	
** >10 years**	24.85	20.72	
** Unclassified**	58.71	71.25	
**Education level (%)**			<0.001
** < High school**	21.47	13.99	
** High school**	34.27	22.45	
** >High school**	44.21	63.48	
** Unclassified**	0.05	0.08	
**Annual family income (%)**			<0.001
** < 20000 USD**	34.26	13.16	
** ≥20000 USD**	60.89	81.48	
** Unclassified**	4.85	5.36	
**Chronic bronchitis**	41.93	5.41	<0.001
**Emphysema**	35.31	0.62	<0.001
**Asthma (%)**	40.68	12.45	<0.001
**Diabetes (%)**	29.54	15.02	<0.001
**Chronic heart failure (%)**	18.76	2.83	<0.001
**Coronary heart disease (%)**	23.49	4.70	<0.001
**Stroke (%)**	14.21	3.91	<0.001
**Lung cancer**	3.39	0.14	<0.001

Mean ± SD for continuous variables, p-value was calculated by weighted linear regression model; % for categorical variables, p-value was calculated by weighted chi-square test; NHANES, National Health and Nutrition Examination Survey; COPD, chronic obstructive pulmonary disease.

### Association between COPD and risk of lung cancer

**Fig 2:**
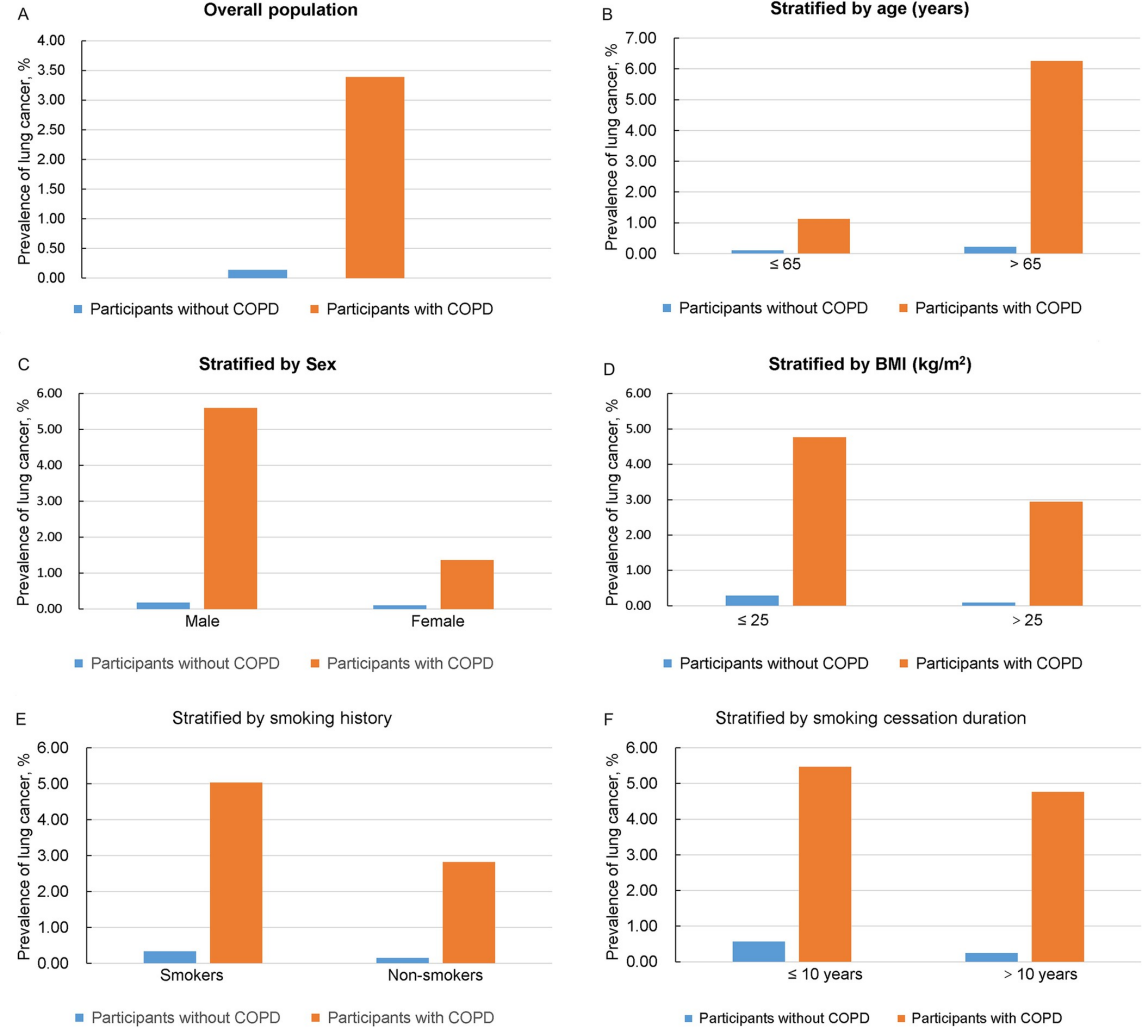
Comparison of prevalence of lung cancer in participants with COPD and participants without COPD. (A) In overall population. (B) Stratified by age. (C) Stratified by sex. (D) Stratified by BMI. (E) Stratified by smoking history. (F) Stratified by smoking cessation duration. COPD, chronic obstructive pulmonary disease; BMI, body mass index.

**Fig 3:**
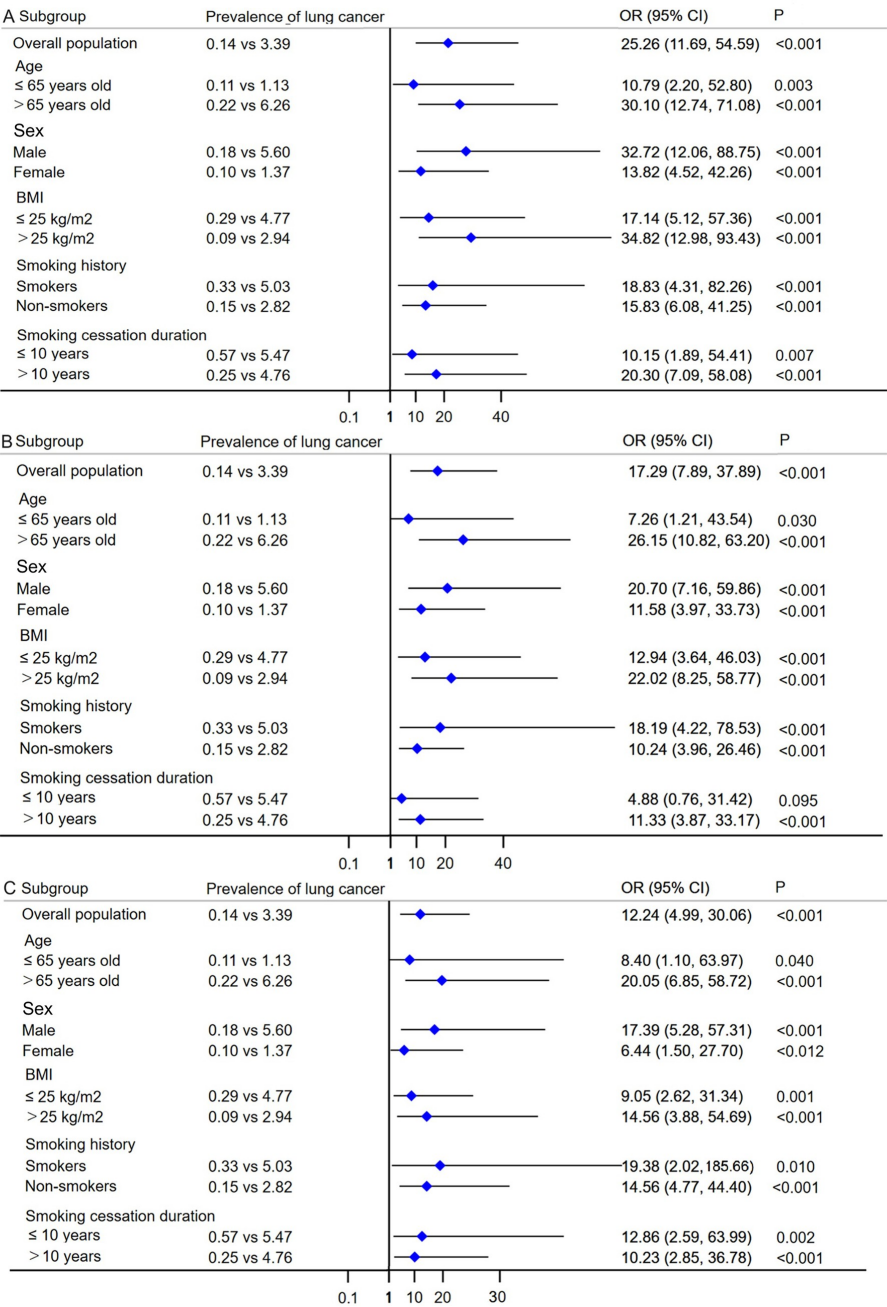
Forest plot showing association of COPD with prevalence of lung cancer. (A) Unadjusted model: with no covariate adjustment; (B) Minimally adjusted model: with adjustment of age, sex, and race; (C) Fully adjusted model: with adjustment of age, sex, race, BMI, education level, annual family income, smoking, comorbidities (including asthma, diabetes, chronic heart failure, coronary heart disease, stroke). COPD, chronic obstructive pulmonary disease; BMI, body mass index.

The prevalence of lung cancer in participants with COPD was significantly higher than that in participants without COPD (3.39% vs 0.14%), and this difference was consistent across all subgroups and different cycles of NHANES (**Fig 2**; **S1 Fig in S1 File**). The weighted multivariable logistic regression models showed that COPD was significantly associated with an increased risk of lung cancer in all three models: (1) unadjusted model with no covariate adjustment: (OR = 25.26, 95% CI = 11.69, 54.59, p < 0.001); (2) minimally adjusted model with adjustment for age, sex, and race: (OR = 17.29, 95% CI = 7.89, 37.89, p < 0.001); fully adjusted model with adjustment for all analyzed covariates, including baseline demographic characteristics, lifestyle factors, and comorbidities: (OR = 12.24, 95% CI = 4.99, 30.06, p < 0.001) (**Table 2** and **Fig 3**).

**Table 2:** Comparison of risk of lung cancer in participants with COPD and participants without COPD.

Participants	Lung cancer N (%)		Risk of lung cancer: OR (95% CI) *P* value		
	Participants without COPD	Participants with COPD	Unadjusted OR	Minimally adjusted OR	Fully adjusted OR
**Overall**	0.14	3.39	25.26 (11.69, 54.59) <0.001	17.29 (7.89, 37.89) <0.001	12.24 (4.99, 30.06) <0.001
**Age**					
**≤ 65 years old**	0.11	1.13	10.79 (2.20, 52.80) 0.003	7.26 (1.21, 43.54) 0.030	8.40 (1.10, 63.97) 0.040
**>65 years old**	0.22	6.26	30.10 (12.74, 71.08) <0.001	26.15 (10.82, 63.20) <0.001	20.05 (6.85, 58.72) <0.001
**Sex**					
**Male**	0.18	5.60	32.72 (12.06, 88.75) <0.001	20.70 (7.16, 59.86) <0.001	17.39 (5.28, 57.31) <0.001
**Female**	0.10	1.37	13.82 (4.52, 42.26) <0.001	11.58 (3.97, 33.73) <0.001	6.44 (1.50, 27.70) 0.012
**BMI**					
**≤ 25 kg/m** ^**2**^	0.29	4.77	17.14 (5.12, 57.36) <0.001	12.94 (3.64, 46.03) <0.001	9.05 (2.62, 31.34) 0.001
>**25 kg/m**^**2**^	0.09	2.94	34.82 (12.98, 93.43) <0.001	22.02 (8.25, 58.77) <0.001	14.56 (3.88, 54.69) <0.001
**Smoking**					
**Smoking history**					
**Smokers**	0.33	5.03	18.83 (4.31, 82.26) <0.001	18.19 (4.22, 78.53) <0.001	19.38 (2.02, 185.66) 0.010
**Non-smokers**	0.15	2.82	15.83 (6.08, 41.25) <0.001	10.24 (3.96, 26.46) <0.001	14.56 (4.77, 44.40) <0.001
**Smoking cessation duration**					
**≤ 10 years**	0.57	5.47	10.15 (1.89, 54.41) 0.007	4.88 (0.76, 31.42) 0.095	12.86 (2.59, 63.99) 0.002
**>10 years**	0.25	4.76	20.30 (7.09, 58.08) <0.001	11.33 (3.87, 33.17) <0.001	10.23 (2.85, 36.78) <0.001

Unadjusted OR: with no covariate adjustment; Minimally adjusted OR: with adjustment of age, sex, and race, Fully adjusted OR: with adjustment of age, sex, race, BMI, education level, annual family income, smoking, comorbidities (including asthma, diabetes, chronic heart failure, coronary heart disease, stroke). COPD, chronic obstructive pulmonary disease; OR, odds ratio; BMI, body mass index.

### Age-stratified lung cancer risk

In participants **≤** 65 years old, the prevalence of lung cancer was 1.13% and 0.11% for patients with COPD and participants without COPD, respectively. In participants > 65 years old, the prevalence of lung cancer was 6.26% and 0.22% for patients with COPD and participants without COPD, respectively (**Fig 2**). The weighted multivariable logistic regression models showed that COPD was significantly associated with an increased risk of lung cancer regardless of the participant’s age. In the fully adjusted model: (1) **≤** 65 years old: (OR = 8.40, 95% CI = 1.10, 63.97, p = 0.040); (2) > 65 years old: (OR = 20.05, 95% CI = 6.85, 58.72, p < 0.001) (**Table 2 and Fig 3**).

### Sex-stratified lung cancer risk

In male participants, the prevalence of lung cancer was 5.60% and 0.18% for patients with COPD and participants without COPD, respectively. In female participants, the prevalence of lung cancer was 1.37% and 0.10% for patients with COPD and participants without COPD, respectively (**Fig 2**). The weighted multivariable logistic regression models showed that COPD was significantly associated with an increased risk of lung cancer regardless of the participant’s sex. In the fully adjusted model: (1) male: (OR = 17.39, 95% CI = 5.28, 57.31, p < 0.001); (2) female: (OR = 6.44, 95% CI = 1.50, 27.70, p = 0.012) (**Table 2** and **Fig 3**).

### BMI stratified lung cancer risk

In participants with a BMI **≤** 25 kg/m^2^, the prevalence of lung cancer was 4.77% for patients with COPD and 0.29% for participants without COPD. In participants with a BMI > 25 kg/m^2^, the prevalence of lung cancer was 2.94% for patients with COPD and 0.09% for participants without COPD (**Fig 2**). The weighted multivariable logistic regression models showed that regardless of the participant’s BMI, COPD was significantly associated with an increased risk of lung cancer. In the fully adjusted model: (1) BMI **≤** 25 kg/m^2^: (OR = 9.05, 95% CI = 2.62, 31.34, p = 0.001); (2) BMI ≥ 25 kg/m^2^: (OR = 14.56, 95% CI = 3.88, 54.69, p < 0.001) (**Table 2** and **Fig 3**).

### Smoking status stratified lung cancer risk

In smokers, the prevalence of lung cancer was 5.03% for patients with COPD and 0.33% for participants without COPD. In non-smokers, the prevalence of lung cancer was 2.82% for patients with COPD and 0.15% for participants without COPD (**Fig 2**). The weighted multivariable logistic regression models showed that regardless of the participant’s smoking status, COPD was significantly associated with an increased risk of lung cancer. In the fully adjusted model: (1) smokers: (OR = 19.38, 95% CI = 2.02, 185.66, p = 0.010); (2) non-smokers: (OR = 14.56, 95% CI = 4.77, 44.40, p < 0.001) (**Table 2** and **Fig 3**).

In participants with a smoking cessation duration ≤ 10 years, the prevalence of lung cancer was 5.47% for patients with COPD and 0.57% for participants without COPD. In participants with a smoking cessation duration > 10 years, the prevalence of lung cancer was 4.76% for patients with COPD and 0.25% for participants without COPD (**Fig 2**). The weighted multivariable logistic regression models showed that regardless of the participant’s smoking cessation duration, COPD was significantly associated with an increased risk of lung cancer. In the fully adjusted model: (1) smoking cessation duration ≤ 10 years: (OR = 12.86, 95% CI = 2.59, 63.99, p = 0.002); (2) smoking cessation duration > 10 years: (OR = 10.23, 95% CI = 2.85, 36.78, p < 0.001) (**Table 2** and **Fig 3**).

## Discussion

Compared to patients without COPD, the proportion of older participants, smokers, smoking cessation duration ≤ 10 years, those with lower educational levels and lower annual family incomes, and those with chronic cardiopulmonary comorbidities was higher among patients with COPD. The prevalence of lung cancer in patients with COPD was significantly higher than that in participants without COPD. And the difference in the lung cancer prevalence was consistent across all subgroups and different cycles of NHANES. After adjusting for multiple potential confounding factors, including baseline demographic characteristics, lifestyle, and comorbidities, particularly the influence of smoking, there was still a significant association between COPD and the increased risk of lung cancer. The results of subgroup analyses were consistent with the overall conclusion, suggesting that COPD was an independent risk factor for increased lung cancer risk, independent of age, sex, BMI, and smoking status of the patients. In certain subgroups, the increased risk of lung cancer is more prominent, including individuals aged > 65 years, smokers, males, individuals who quit smoking within 10 years, and individuals with a BMI > 25 kg/m^2^. In addition, the combination of COPD and smoking greatly amplifies the lung cancer risk. This is evident in the fact that both smokers with and without COPD have a higher risk of developing lung cancer compared to non-smokers. However, quitting smoking for over ten years may reduce the lung cancer risk. To the best of our knowledge, this is the first cross-sectional study to found that the combination of COPD and smoking significantly amplifies the risk of developing lung cancer.

The possible mechanisms by which COPD leads to lung cancer include chronic inflammation and oxidative stress caused by COPD, which can lead to DNA damage and mutations, increasing the likelihood of cell carcinogenesis. In addition, the damaged lung tissue in patients with COPD may release inflammatory mediators and growth factors that stimulate the growth and division of lung cancer cells [21–23]. Moreover, the lung tissue of patients with COPD is already damaged by inflammation and oxidative stress, making it more vulnerable to other carcinogens such as smoke and toxic chemicals [7, 21]. Glucocorticoids may have a protective effect against lung cancer. Dexamethasone has been shown to exert growth-inhibitory effects on several glucocorticoid receptor (GR) rich NSCLC cell lines [24, 25]. This mechanism may be due to glucocorticoids reducing airway, pulmonary, and systemic inflammation in patients with COPD [3, 26]. Additionally, glucocorticoids may inhibit proto-oncogenes in human smokers by modulating the COX-2 inflammatory pathway, which is overexpressed in most NSCLCs and plays a role in tumor progression [27–29]. Similarly, the study by Woo et al. [30] suggests that among patients with asthma, a higher cumulative dose of inhaled corticosteroids (ICS) is associated with a 56% reduction in lung cancer risk, further supporting the potential protective role of glucocorticoids against lung cancer.

Our results support previous studies on the increased risk of lung cancer in patients with COPD [12–14]. In addition, our study provides new evidence that COPD is an independent risk factor for lung cancer. Our study shows that, even after adjusting for the influence of covariates, especially smoking, the risk of developing lung cancer in patients with COPD remains significantly increased compared to patients without COPD. Previous studies have shown that COPD is associated with poor survival prognosis and a higher risk of recurrence in early-stage lung cancer) [31]. A retrospective study conducted by Zhai et al. suggested that the 5-year OS and PFS rates of lung cancer patients with COPD were significantly lower than those without COPD (OS rate: 54.4% vs. 69.0%, PFS rate: 50.1% vs. 60.6%) [31]. Therefore, attention should be paid to lung cancer screening in patients with COPD to avoid treatment delays. Additionally, it is worth noting that the co-management of COPD in lung cancer patients needs urgent clinical attention.

It should be noted that the causes of COPD are complex, including smoking, air pollution, occupational dust exposure, genetics, etc. Therefore, patients with COPD have different clinical phenotypes due to different etiology. Our subgroup analyses results suggested that the risk of developing lung cancer is higher in patients with COPD who are aged > 65 years, smokers, males, individuals who quit smoking within 10 years, and individuals with a BMI > 25 kg/m^2^, compared to those who are aged ≤ 65 years, non-smokers, females, individuals who quit smoking over 10 years, and individuals with a BMI ≤ 25 kg/m^2^. Considering that early detection of lung cancer is crucial for successful treatment, patients with COPD in the high-risk subgroups should be subjected to more active screening for lung cancer to facilitate early diagnosis and treatment. Furthermore, our subgroup analyses revealed a synergistic effect between smoking and COPD in increasing the risk of lung cancer, further highlight the importance of smoking cessation for patients with COPD. Further research focusing on predictive factors related to lung cancer susceptibility in patients with COPD is needed to provide more evidence.

Our study must be distinguished from another study conducted by Rahman et al [32] based on NHANES, which investigated the association between lung diseases (including COPD) and lung cancer risk. There are several differences in study design and findings between the two studies. Firstly, the objectives of the studies differ. We assessed the lung cancer risk in populations with different characteristics (such as age, sex, BMI, smoking) comparing individuals with COPD to those without COPD, i.e., COPD vs non-COPD on lung cancer risk. In contrast, their study assessed the association between various covariates (age, sex, etc.) and lung cancer in patients with COPD, such as male COPD vs female COPD on lung cancer risk. Secondly, the study populations differ. Our study included individuals aged 40 and older, as it is widely accepted that COPD primarily occurs in this age group. COPD diagnosed in individuals under 40 may be challenging to distinguish from asthma and could introduce bias into the study results [33, 34]. However, their study did not exclude individuals under 40. Thirdly, our study had a larger sample size. We included data from three cycles of NHANES, including 660 patients with COPD, whereas their analysis included only 165 patients with COPD. Therefore, our results may be more representative. Additionally, considering that common comorbidities in older adults may be important confounding factors in the association between COPD and lung cancer [35], we adjusted for multiple comorbidities in the weighted multivariable logistic regression models, while their study did not adjust for comorbidity effects. Finally, to eliminate the influence of smoking and confirm that COPD is an independent risk factor for lung cancer, we conducted subgroup analyses based on patients’ smoking history and smoking cessation duration.

Our study has a significant advantage in that we utilized data from NHANES 2013–2018. The NHANES database follows standardized protocols for participant information collection, with a large nationally representative sample size. This means that our findings have broad applicability. Additionally, our study’s multiple subgroup analyses consistently support our primary outcome, lending further support to the reliability of our conclusions. However, our study does have some limitations. First, due to its cross-sectional design, our results can only establish an association between COPD and lung cancer, rather than a causal relationship. Second, the NHANES dataset did not include detailed information on the severity of COPD, lung cancer stage, and histological cell type, which further limits our ability to analyze the relationship between COPD and lung cancer in a more in-depth and accurate manner with these factors. Future studies incorporating these details is necessary to gain a fuller understanding of the relationship between COPD and lung cancer. Furthermore, smoking plays a pivotal role in the etiology of lung cancer, with smoking-related factors such as cumulative smoking exposure and passive smoking significantly influencing the lung cancer risk in patients with COPD. Indeed, our research solely relies on NHANES data, limiting our further subgroup analyses on the association between smoking and lung cancer risk from various perspectives due to lack of data. Therefore, more prospective studies are needed in the future. Third, the NHANES dataset lacks information on environmental/occupational exposure related to lung cancer in patients with COPD, which could have provided valuable insights into how environmental/occupational factors contribute to lung cancer risk. Future research will need to address this gap by incorporating environmental/occupational exposure data to further elucidate its role in lung cancer risk among patients with COPD. Fourth, the diagnosis of COPD was based on self-reported questionnaire data, which may be subject to recall bias and misclassification. Additionally, about 40% of patients with COPD in our study also have asthma, that is, patients with asthma-COPD overlap syndrome (ACOS), which presents distinct clinical characteristics compared to COPD without asthma. The study by Woo et al. [30] indicates that asthma development is linked to increased cancer risk, while another study by Charokopos et al. [36] reports that patients with ACOS have a lung cancer risk similar to that of patients with COPD alone. Further studies are needed to clarify the lung cancer risk in these distinct groups, including COPD, ACOS, and asthma patients. Finally, the study population from the NHANES is predominantly composed of Non-Hispanic White and Non-Hispanic Black individuals from the United States, and as such, careful consideration should be taken when generalizing our findings to other populations.

## Conclusion

In conclusion, COPD is an independent risk factor for lung cancer, especially in certain subgroups including individuals aged > 65 years, smokers, males, individuals who quit smoking within 10 years, and individuals with a BMI > 25 kg/m^2^. The combination of COPD and smoking greatly amplifies the lung cancer risk. These findings highlight the importance of early lung cancer screening in patients with COPD.

## Supporting information

S1 File (DOCX)
