# Supplementary material for: Association of chronic obstructive pulmonary disease with risk of lung cancer in individuals aged 40 years and older: A cross-sectional study based on NHANES 2013–2018
Source: PLoS One. 2024 Oct 23;19(10):e0311537. doi: 10.1371/journal.pone.0311537 (PMC11498685; doi:10.1371/journal.pone.0311537)
Supplement: S1 File — (DOCX) [file pone.0311537.s001.docx]

**Supplementary Material**

**Table S1: Data source for** **exposure variable, outcome variable, and covariates**

| **Variables** | **Questionnaire items** | **Data source: NHANES questionnaire data (Medical Conditions section)** |
| --- | --- | --- |
| **Exposure variable** | | |
| **COPD** | MCQ160o - Ever told you had COPD? | Questionnaire Data (Medical Conditions section): the researchers surveyed the participants about whether they had received a medical diagnosis of COPD from a healthcare professional. Participants who answered “yes” were assigned to the COPD group, while those who replied “No” were assigned to the control group. Participants with missing data on this question were excluded from this study. |
| **Outcome variable** | | |
| **Lung cancer** | MCQ240n - Age when lung cancer first diagnosed | Questionnaire Data (Medical Conditions section): the researchers surveyed the participants about whether they had received a medical diagnosis of lung cancer from a healthcare professional. Participants who answered “yes” were assigned to the COPD group, while those who replied “No” were assigned to the control group. Participants with missing data on this question were excluded from this study. |
| **Other** | | |
| **Demographic data** | | |
| **Age** | —— | Demographics Data |
| **Sex** | —— | Demographics Data |
| **Race** | —— | Demographics Data |
| **BMI** | —— | Examination Data (Body Measures section) |
| **Smoking related data** | SMQ040 - Do you now smoke cigarettes? | Questionnaire Data (Smoking - Cigarette Use section) |
| **Socioeconomic status** | | |
| **Annual family income** | —— | Demographics Data |
| **Education level** | —— | Demographics Data |
| **Common comorbidities** | | |
| **Chronic bronchitis** | MCQ160k - Ever told you had chronic bronchitis | Questionnaire Data (Medical Conditions section): the researchers surveyed the participants about whether they had received a medical diagnosis of chronic bronchitis from a healthcare professional |
| **emphysema** | MCQ160g - Ever told you had emphysema | Questionnaire Data (Medical Conditions section): the researchers surveyed the participants about whether they had received a medical diagnosis of emphysema from a healthcare professional |
| **Asthma** | MCQ010 - Ever been told you have asthma | Questionnaire Data (Medical Conditions section): the researchers surveyed the participants about whether they had received a medical diagnosis of asthma from a healthcare professional. |
| **Diabetes** | DIQ010 - Doctor told you have diabetes | Questionnaire Data (Diabetes section): the researchers surveyed the participants about whether they had received a medical diagnosis of diabetes from a healthcare professional. |
| **Chronic heart failure** | MCQ160b - Ever told had congestive heart failure | Questionnaire Data (Medical Conditions section): the researchers surveyed the participants about whether they had received a medical diagnosis of chronic heart failure from a healthcare professional. |
| **Coronary heart disease** | MCQ160c - Ever told you had coronary heart disease | Questionnaire Data (Medical Conditions section): the researchers surveyed the participants about whether they had received a medical diagnosis of coronary heart disease from a healthcare professional. |
| **Stroke** | MCQ160f - Ever told you had a stroke | Questionnaire Data (Medical Conditions section): the researchers surveyed the participants about whether they had received a medical diagnosis of stroke from a healthcare professional. |

All data were extracted from NHANES 2013-2018 (consisting of 3 cycles of NHANES, including NHANES 2013-2014, NHANES 2015-2016, and NHANES 2017-2018) (http://www.cdc.gov/nchs). NHANES National Health and Nutrition Examination Survey, COPD chronic obstructive pulmonary disease

**Figure S1: Comparison of prevalence of lung cancer in participants with COPD and participants without COPD in different NHANES cycles**


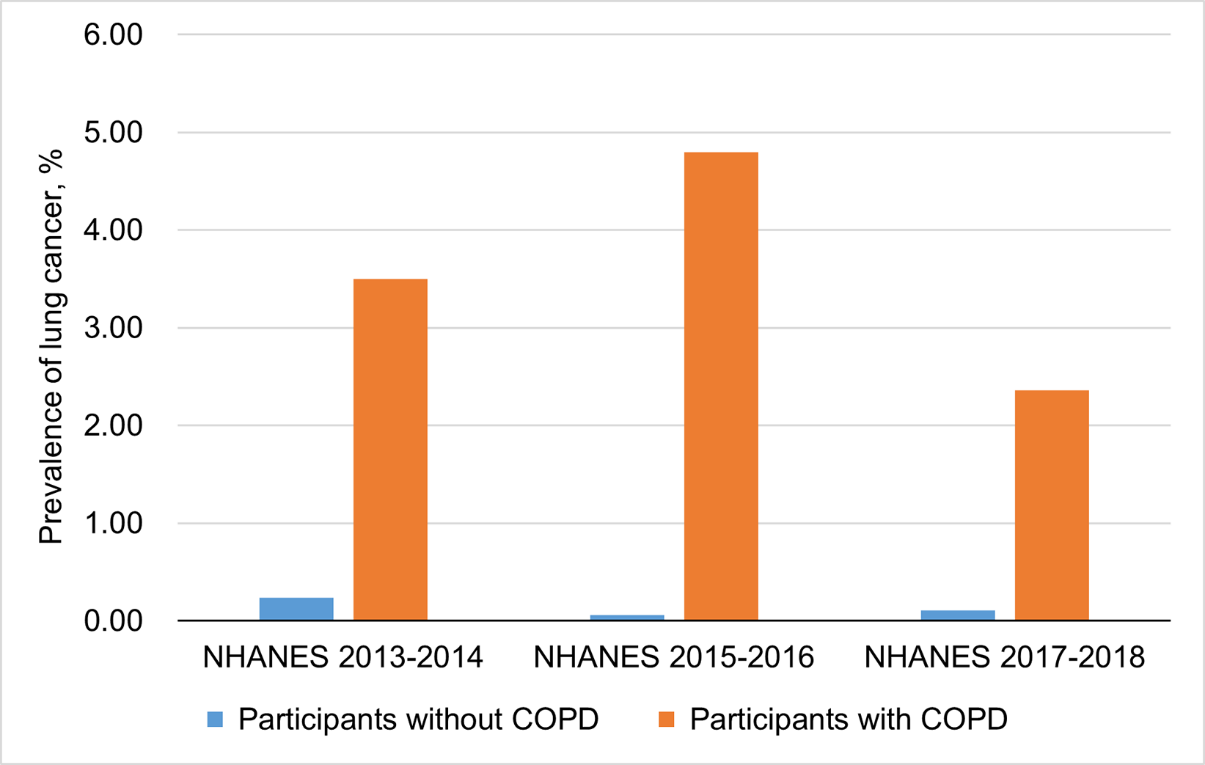
NHANES National Health and Nutrition Examination Survey, COPD chronic obstructive pulmonary disease
